# A multicenter, randomized controlled trial of massage in children with pediatric cerebral palsy: Efficacy of pediatric massage for children with spastic cerebral palsy

**DOI:** 10.1097/MD.0000000000023469

**Published:** 2021-02-05

**Authors:** Can Zhang, Guangyi Xiong, Jian Wang, Xinan Shi, Taipin Guo, Yaju Jin, Yan Zhao, Xiantao Tai

**Affiliations:** a First Clinical Medical College, Nanjing University of Chinese Medicine, 282 Hanzhong Road, Gulou, Nanjing, Jiangsu; b Medical ethics committee of the second affiliated hospital of Yunnan University of Chinese Medicine; c School of Basic Medicine, Yunnan University of Chinese Medicine, 1076 Yuhua Road, Chenggong District, Kunming, Yunnan, China.

**Keywords:** pediatric massage, randomized controlled trial, spastic cerebral palsy

## Abstract

**Background:**

Cerebral palsy is 1 of the diseases critically affecting the health of children. The spasmodic type is the most common, characterized by the increased muscular tension. It often leads to lifelong disability, bringing a heavy economic burden to families and society. As a key treatment in traditional Chinese medicine, pediatric massage has a significant clinical effect on cerebral palsy in children; however, high-quality randomized controlled studies are lacking. The main objective of this study was to evaluate the efficacy of pediatric massage for children with spastic cerebral palsy.

**Methods/Design:**

The study will be a multicenter, single-blinded, randomized-controlled pilot trial. During the period from June 2019 to December 2020, 182 children with spastic cerebral palsy will be randomly divided into experimental and control groups in a 1:1 ratio. The experimental group will undergo the modified selective spinal massage method combined with the basic rehabilitation treatment, while only the basic rehabilitation treatment would be performed for the control group. The intervention period of the study will last 12 weeks, 5 days weekly on weekdays. The primary outcomes include a modified Ashworth scale assessment and gross motor function test. The secondary outcomes include the 4-diagnostic scale of Chinese medicine and children's intelligence. The observation index will be measured during the complete 12 weeks duration after the treatment of the child, that is, before treatment, after 4 weeks of treatment, after 8 weeks, and after 12 weeks of treatment.

**Discussion:**

This study aims to evaluate the efficacy of pediatric massage on children with spastic cerebral palsy; if the outcome is positive, it can provide a reference for the further promotion and application of pediatric massage in the treatment of spastic cerebral palsy.

**Trial registration:**

Chinese ClinicalTrials.gov, ID: ChiCTR1900021666. Acupuncture-Moxibustion Clinical Trial Registry, AMCTR: (AMCTR-IPR-19000260) Registered on 04 March 2019.

## 1 Introduction

Cerebral palsy describes a set of permanent diseases that develop in motion and posture, leading to limited activity, owing to the occurrence of non-progressive disorders in the developing fetuses or the baby's brain. The dyskinesia of cerebral palsy is usually accompanied by disturbances in perception, cognition, communication, behavior, epilepsy, and secondary musculoskeletal problems.^[1]^ Cerebral palsy is 1 of the major diseases causing disability in children. Currently, the prevalence rate of cerebral palsy is approximately 1.5% to 4% worldwide and 2.48% in China, among which the spastic type is the most common, accounting for about 60%.^[2,3]^ It is mainly characterized by increased muscle tension. Studies have shown that the most common type of cerebral palsy in premature infants is spastic diplegia, accounting for 52.7%.^[4]^ The disease with a high incidence is harmful and is 1 of the important reasons for harming the health of children. It often leads to permanent disability, seriously affects the quality of life and mental health of children, and brings a heavy economic burden to families and society.

Currently, there is no specific medication for the treatment of spastic cerebral palsy. The most commonly used are anti-muscular tension drugs. Most of the anti-spasmodic drugs, including benzodiazepines and baclofen, can be used in combination, which can reduce muscle tension, relieve spasm, and improve function. However, side effects such as ataxia, constipation, drowsiness, and dependence commonly occur in clinical practice.^[5]^ Although botulinum toxin can relieve the spasm quickly, its effect is short-lasting and requires repeated treatment, which aggravates the financial burden on the patient's family.^[6]^ Non-drug therapy mainly includes surgical treatment and rehabilitation training. Selective posterior rhizotomy of the spinal nerve is a newer surgical method for the treatment of spasmodic cerebral palsy and is effective in alleviating spasmodic state.^[7]^ However, there are certain requirements for patients. The optimal age of operation is 2 to 6 years, and it is appropriate only for patients with spastic cerebral palsy, close to normal intelligence, with muscle tension above grade 3, and maintaining certain muscle strength and motor function.^[8]^ Patients with hand-foot-crawling and ataxia types of cerebral palsy are not suitable for this operation. Postoperative rehabilitation training is a fundamental requirement for successful treatment. Surgery also causes trauma, and a high recurrence rate is observed during follow-up; therefore, the choice of surgical treatment should be carefully considered.^[9]^ Rehabilitation training is the most widely used treatment for cerebral palsy. The common rehabilitation methods include physical therapy, occupational therapy, exercise therapy, speech training, sensory training, and guided education.

The pediatric massage is based on the basic theory of Traditional Chinese Medicine, the practices on the meridian and acupoints, giving the body a benign physical stimulation, making the function of zang-fu organs blood of equilibrium state and is economical, convenient, and safe. It has the advantages of easy acceptance for improving cerebral palsy. Clinical studies^[10–12]^ have shown that massage is effective in the motor development, sleep quality and learning, and memory effects of cerebral palsy. The evidence for most of these interventions is weak, partly owing to the lack of randomized controlled trials; however, also because of the many confounding factors of treatment, such as the effects of comorbidities. Therefore, we propose that massage therapy can improve the functional recovery of children and help in designing the randomized controlled trials to further improve the relevant evidence.

## 2 Methods

### 2.1 Study design

The proposed study will be a multicenter, single-blinded, randomized controlled pilot trial. The trial will be commenced after ethical approval has been obtained from the second affiliated hospital of the Yunnan University of Chinese Medicine. The clinical trial will be conducted in the Yunnan rehabilitation center for the disabled, Kunming children's hospital, the second affiliated hospital of the Yunnan University of traditional Chinese medicine, and Yuxi Hospital of Traditional Chinese medicine. All study-related procedures will be performed after obtaining written informed consent from the participants. The clinical trial is designed and reported following the Consolidated Standards of Reporting Trials guidelines.

### 2.2 Participants

#### 2.2.1 Sample size estimation

The estimated sample size will be compared using 2 sets of odds ratios. The formula is as follows:

$$n_{1}={\left[\frac{z_{1}-\alpha /2\sqrt{\bar{p}\bar{q}(1+1/r)}+z_{1}-\beta \sqrt{p_{1}q_{1}+p_{2}q_{2}/r}}{\Delta }\right]}^{2}=82$$

A total of 182 children were calculated using the transcript estimation method for the robustness of the clinical trial. The specific index is that α = 0.05 on both sides of the tail, and (1-β) is 0.80, and the absolute difference of the comprehensive result variable index is 20%. Our initial estimate of the sample size is 82, and the loss rate is increased by 10%, and 91 is determined for each group. Thus, the 2 groups totaled 182.^[13,14]^

### 2.3 Subject recruitment

Inclusion criteria:

(1) Aged 1 to 3 years, both male and female children.
(2) Grading of the large motor function at the I-III level.^[15,16]^
(3) Children who meet the diagnostic criteria for spastic cerebral palsy.^[17]^
(4) Muscle tone between I-IV (according to the newly revised Ashworth scale).
(5) Follow the doctor advice during the study and insist on receiving the tester.

Exclusion criteria:

(1) Those with severe mental illness and severe epilepsy.
(2) Organized diseases involving other organs such as the heart, liver, and kidney.
(3) Exclusion of leukodystrophy, infantile spinal muscular atrophy, spin cerebellar ataxia, genetic metabolic diseases such as phenylketonuria, congenital cretinism, and congenital hydrocephalus.
(4) Failure to meet the inclusion criteria, failure to complete the test according to the regulations, and the treatment of patients who have an influence on the test results during the study, the incomplete data affect the clinical efficacy observation.
(5) Spontaneous bleeding tendency.

### 2.4 Randomization and blinding

**Figure 1:**
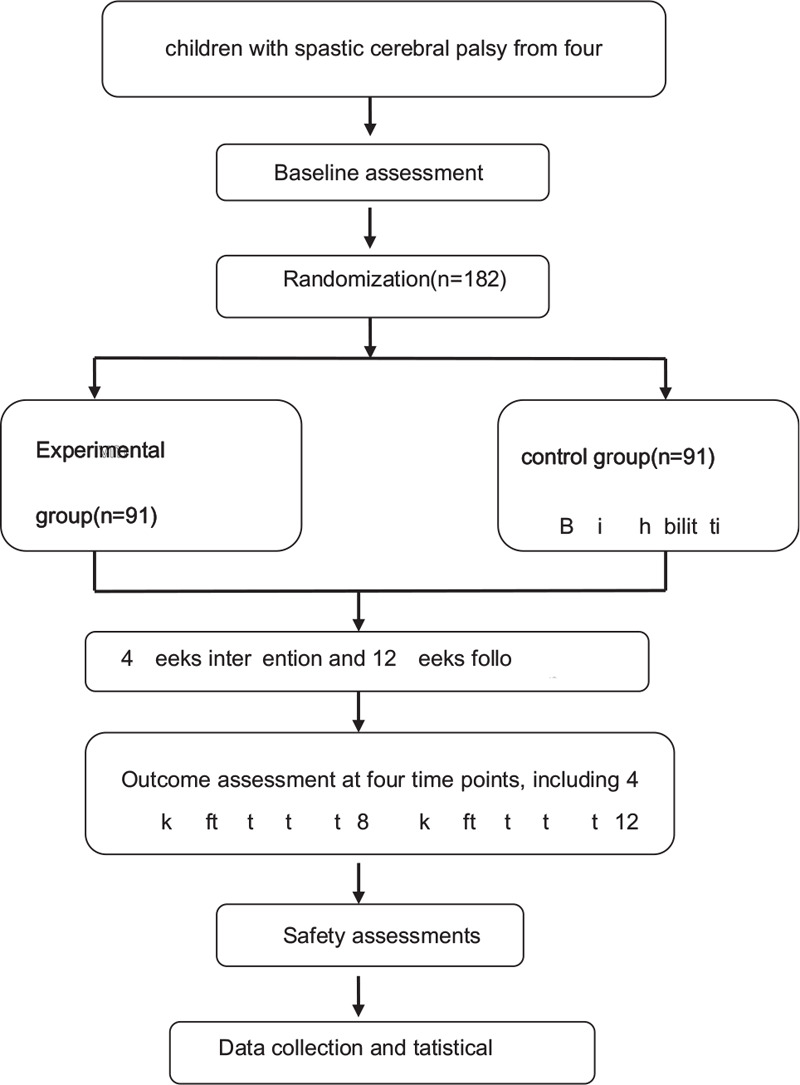
Flow diagram of the study design.

The staff member will refer to a specific random number handbook generated by an independent statistician based on statistical software before study activation to determine the study intervention assigned to the randomized patient. The randomization schedule is layered by the position of each 4-bit variable block. Participants will be randomly assigned to 1 of the 2 groups in a 1:1 ratio after completion of baseline assessment, according to a randomized block list generated by an independent research assistant using R, and the code will be kept in opaque sequentially numbered envelopes. The treatment allocation codes will be enclosed by an independent research assistant in sequentially numbered opaque envelopes and will not be revealed until the participants have completed all baseline assessments and immediately before the first massage treatment. The single-blind method is adopted in this study, and there are two-level blind methods. First, blind evaluation, in which the participants are evaluated by a third party who does not know the grouping. Second, the blind statistical analysis, where the statistical data will be analyzed by statisticians with unclear grouping and its meaning. (Fig. 1)

### 2.5 Treatment protocol

1. Experimental group

1) Pediatric massage method:^[18]^

Place the child in a quiet, warm treatment room at room temperature between 25°C and 28°C. Appease the child before the operation to keep him in a quiet state.

Steps:②

Stimulation Governor Vessel and The Bladder Meridian of Foot-Taiyang. The child will take the prone position, the doctor will stand on the right side, and will use the Mofa along Governor Vessel direction to rub the entire spine, from top to bottom 3 to 5 times; Anrou the back of the first sideline and the second sideline of The Bladder Meridian of Foot-Taiyang by up to down 3 to 5 times; pinch the spine from top to bottom 3- 5 times; rubbing Shenshu (BL23), Mingmen (DU4), and Baliao.②

According to the principle of spinal neuroanatomy, the projection point on the surface of the spinal nerve root of children with spastic cerebral palsy will be determined, which will usually be the place with a cord or nodal reactants or the position of muscle tension and spasm, and the stimulation will be performed by rubbing for 3 min.③

Local treatment: According to the degree of spasm of the paralyzed limb, local treatment will be carried out using the traditional methods. The child will be placed in a supine position, and the doctor will use rolling to relax limbs, simultaneously, by the passive movement of the corresponding joint. Pressing will be performed to knead paralytic limb in turn, from top to bottom, applying gentle holding method to paralytic limb for 3–5 times. Following this, moderate shaking, pulling, or pulling to the corresponding joints of the paralyzed limb will be applied.④

**Figure 2:**
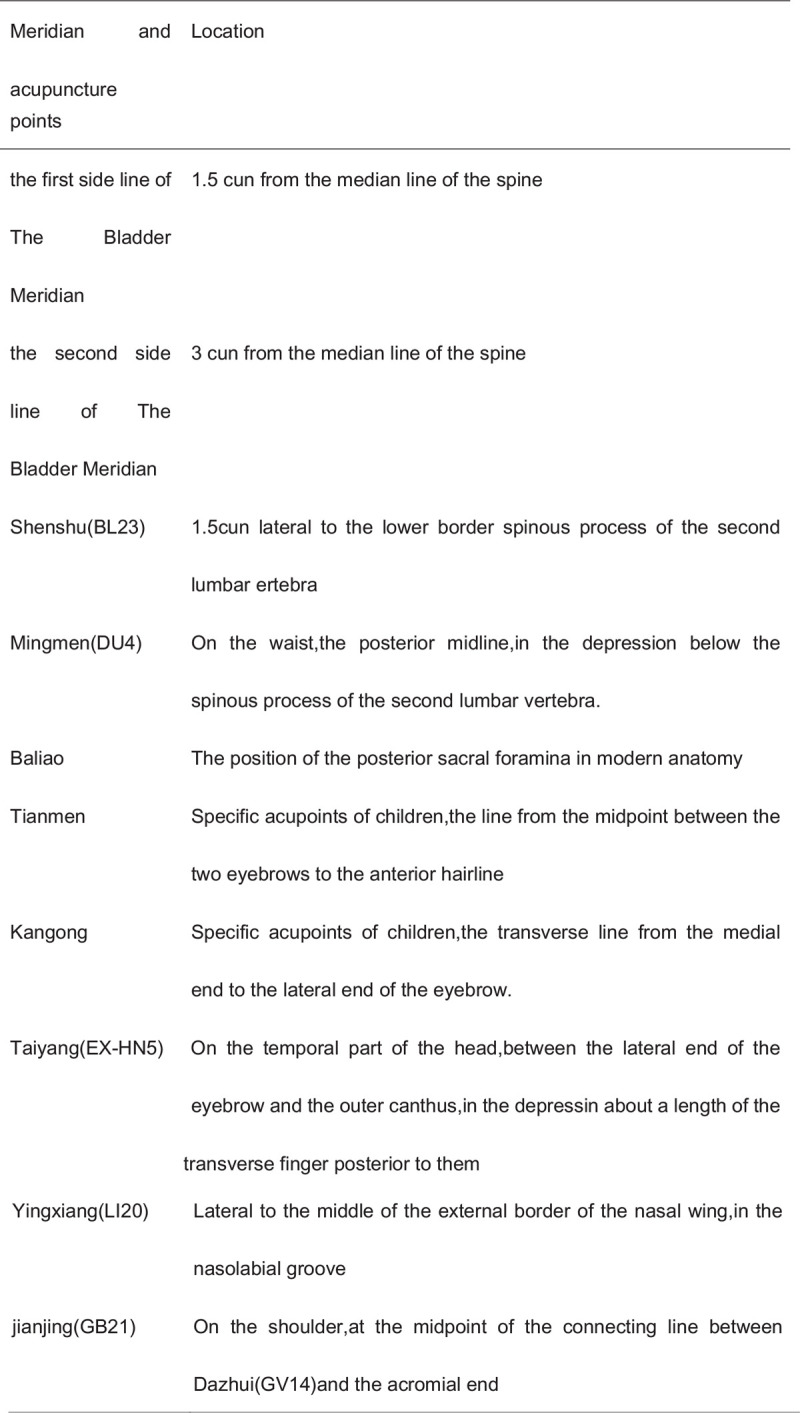
Location of acupoints.

Regulating yin and yang. The child will be placed in the supine position; the doctor will stand on the head side, opening the Tianmen 30 times, separate-pushing the Kangong 30 times, separate-pushing Ying Xiang (LI20) 30 times, kneading Taiyang (EX-HN5) for 1 min, and grasping Jianjing (GB21) 3 to 5 times. (Fig. 2)

(1) Basic rehabilitation treatment group
(2) Treatment Procedure: 1 treatment per day, 5 times a week, 15 to 20 minutes each time, 4 weeks for a course of treatment, a total of 3 courses of treatment. To ensure the scientific and accurate results of the assessment, the assessment will be performed in a single-blinded manner, and all evaluations will be performed by a trained tutor.

(1) Quality assurance of massage treatment
(2) All operators conduct unified training and consistency check. Kappa value is greater than 0.8 to perform the above-mentioned massage operation independently.
(3) Control group: basic rehabilitation treatment group

Basic rehabilitation treatment: All children will receive basic treatment according to the rehabilitation concept applied in the individual hospital and/or as demanded by the cost bearers. It will consist of physical factor therapy, occupational therapy, physiotherapy, and medical exercise.

### 2.6 Measures

#### 2.6.1 Outcome assessment

**Figure 3:**
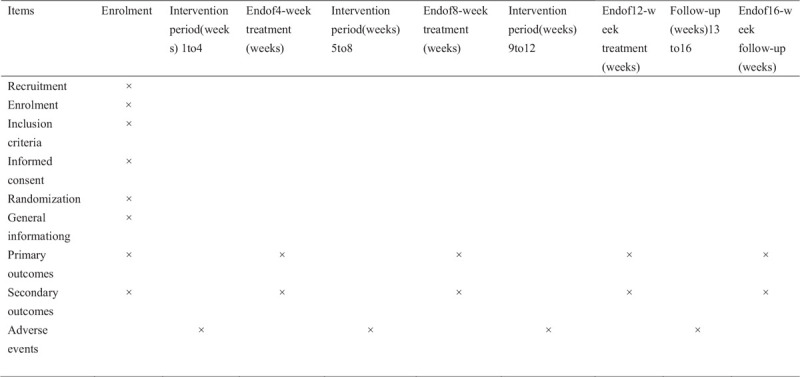
Trial process chart.

All the outcomes will be measured before enrollment, at the end of 4 weeks, the end of 8 weeks, and the end of 12 weeks follow-up. The summer of all the measures in the trial is shown in Figure 3. (Fig. 3)

### 2.7 Primary outcome measure

1. Modified Ashworth Scale
  Owing to the presence of sputum, quadriplegia, and hemiplegia, the lower limb muscle tone (triceps, soleus, gastrocnemius) will be assessed using a modified Ashworth scale.^[19,20]^ It includes 6 rating levels, 0, 1, 1+, 2, 3, and 4. For statistical convenience, 0, 1, 1+, 2, 3, and 4 levels of the muscle tension level in the scale will be determined to be 1, 2, 3, 4, 5, and 6 points, respectively. All subjects are guaranteed not to be treated with any muscle relaxants. During the evaluation, efforts should be made to avoid position metastasis, skin irritation, low ambient temperature, emotional agitation, pain, urinary tract infection, or urination before the examination. If there is a clonic or transient paroxysmal contraction, test again after the disappearance.
2. Gross motor function rating scale (GMFM)

The gross motor function score will be evaluated using the GMFM for gross motor function evaluation. GMFM currently has 88 versions (GMFM-88) and 66 items (GMFM-66).^[21]^ GMFM-88 is a sequential scale, each using a 4-level scoring method, divided into 5 functional areas: Area A (lying position and turning over), a total of 51 points (17 items); Area B (sitting), total score 60 points (20 items); Area C (crawling and squatting), total score of 42 points (14 items); Area D (station), total score 39 points (13 items); Area E (walking, running and jumping). The total score is 72 points (24 items). The evaluation results include 5 functional distinction values and total scores expressing the corresponding energy regions and overall gross exercise capacity, respectively. The Chinese translation is based on the 1993 GMFM revision. Evaluate 3 designated physicians or therapists to perform in a quiet, independent, well-lit room. The room temperature will be controlled at 20°C to 30°C, and the children's clothes will be 1 to 2 layered. In the absence of a violation of the assessment requirements, the same family members should be present in each assessment to encourage the child to perform at an optimal level.

### 2.8 Secondary outcome measures

1. Quantitative measurement of children intelligence-related scales
  The intelligence development scale of Bayley scales of infant development^[22]^ is used to evaluate the intelligence of children with cerebral palsy. It will be independently completed by the special efficacy evaluation team of each hospital.
2. The overall health of children with cerebral palsy

The 4 diagnostic scales of Chinese medicine will be used to record the symptoms of the children, such as diet, sleep, and diarrhea. Regarding the Chinese medicine new medicine clinical research guiding principles^[23]^ and journal of pediatrics of traditional Chinese medicine^[24]^ to develop the 4 clinical observation integral scale of Traditional Chinese Medicine type spasm cerebral palsy, from 4 aspects: inspection, auscultation and olfaction, inquiry, and pulse-taking and palpation of children with the breath, color, shape, state, god, crying, eating, sleeping, mainland, and so on, a comprehensive evaluation of various combined score values (single 2 points, a total of 27 items, maximum 54 points) will be performed.

### 2.9 Safety assessments

All the adverse events (AEs) will be monitored and recorded by researchers throughout the treatment. AEs will be coded using the World Health Organization Adverse Reaction Terminology Dictionary. Skin lesions and rashes are common side effects of massage. Once the AEs occur during the treatment, the time and severity of the treatment will be recorded in detail, and the cause will be analyzed, and the correlation between the AE and trial will be evaluated. If any serious AE occurs, it will be reported to the Human Research Ethics Committee of YNTCM. It will judge whether this child can continue this study. Special attention will be given to those participants who have discontinued treatment owing to AEs or those experiencing serious AEs.

### 2.10 Reasons for withdrawal

When a participant withdraws before completing the study, the reasons for withdrawal will be recorded.

### 2.11 Monitoring

#### 2.11.1 Data and safety monitoring plan

A Data and Safety Monitoring Board will be set up to monitor research progress and review the safety and quality of data. Details of all information will be recorded using the Case Report Form. The committee will consist of 3 members, including a senior massage practitioner, cerebral palsy specialist, and biostatistician. The Data and Safety Monitoring Board is independent of the proposed study, and all the committee members will have to declare any conflict of interest in the study. Regular board meetings will be held to ensure that the data are collected scientifically and ethically, and participants are not exposed to unnecessary risks.

The audit of test conduct will be conducted monthly, and the process will be independent of the investigators and sponsors.

### 2.12 Statistical analyses^[25]^

The primary analysis will be performed using intention-to-treat methods and therefore include all randomized child data. All data will be checked into the double machine using Epidata 3.1 software for database management.

Specific statistical analysis methods: The baseline characteristics of patients in the 2 groups will be reported using frequency distribution and descriptive statistics (including measurements of concentration trends and dispersion). The main analysis of the comprehensive measurement of the rate of counting data will be the chi-square test. Two independent samples of the *t*-test will be performed between the 2 groups. The paired t-test will be used to compare the outcome variables of each treatment group before and after the treatment. The difference between the multiple measurements of the same group will be conducted using the multiple measures data analysis of variance, combined with logistic regression analysis to examine the adjustment effect of clinically relevant covariates, which will be the results (gender, birth weight, gestational age). We will also follow the pre-specified subgroup analysis. All analyses will be performed using the R software. The aforementioned steps will be monitored by the third-party tests, and the quality control of the test will be carried out. The blind law is unified and responsible.

## 3 Discussion

The study protocol of the proposed trial will be conducted at the second affiliated hospital of the Yunnan University of Chinese Medicine. The present study is designed to investigate whether it is feasible, effective, and safe to use massage therapy for pediatric cerebral palsy patients with basic rehabilitation treatment. If successful, the study would provide useful information for integrating massage into rehabilitation care as part of the effort of integrating Chinese and Western medicine in mainland Chinese hospitals. However, this study protocol faces several challenges and limitations.

First, patients with different types of diagnosis of children with cerebral palsy will be included, which may create variability in the population. However, Chinese medicine treatment pays more attention to syndrome differentiation rather than diagnosing disease. Also, for younger children aged <3 years, diagnosis is challenging. Moreover, randomization will be rigorous for balancing the different types of spastic cerebral palsy in the 2 groups.

Second, since this trial only involves the efficacy of massage therapy based on basic rehabilitation therapy, whether massage and basic rehabilitation therapy will form an interaction or massage therapy can be used as an alternative therapy to basic rehabilitation therapy has not been considered.

Third, the quality control of the 4 centers is particularly important, including basic rehabilitation and quality control of massage therapy. Solving this problem relies mainly on continuous training and sincere cooperation of the central treatment team, as well as establishing good relationships with the patient's family to increase compliance and follow-up.

Fourth, the choice of the control group is a difficult point in this study, although this trial is most likely to verify the clinical efficacy of massage therapy.

Currently, we cannot provide an estimate for the massage treatment costs for the treatment and control groups. It is assumed that this study can provide evidence of efficacy and high quality for the rehabilitation of cerebral palsy in children with rehabilitation and useful information for future health economic analysis. We also presume our findings to enhance the knowledge of the effectiveness of massage therapy in the hospital daily comprehensive medical practice.

## 4 Trial status

The first patient was recruited on June 1, 2019, and the last patient will be recruited on December 31, 2020. Patient recruitment is currently being continued

## Author contributions

**Data curation:** Guangyi Xiong, Yaju Jin.

**Formal analysis:** Taipin Guo.

**Funding acquisition:** Xiantao Tai.

**Investigation:** Zhang Can, Taipin Guo, Yan Zhao.

**Methodology:** Yan Zhao, Jian Wang.

**Project administration:** Xiantao Tai.

**Resources:** Xinan Shi.

**Supervision:** Xiantao Tai.

**Writing – original draft:** Zhang Can, Guangyi Xiong.

**Writing – review & editing:** Zhang Can, Xinan Shi.
